# In memoriam: Dr Wan-Kyu Cho, pioneer of developmental biology and architect of Korean science

**DOI:** 10.1016/j.mocell.2026.100388

**Published:** 2026-07-31

**Authors:** Min Whan Jung

**Affiliations:** Center for Synaptic Brain Dysfunctions, IBS / Department of Biological Sciences, KAIST, Daejeon, Korea

**Figure:**
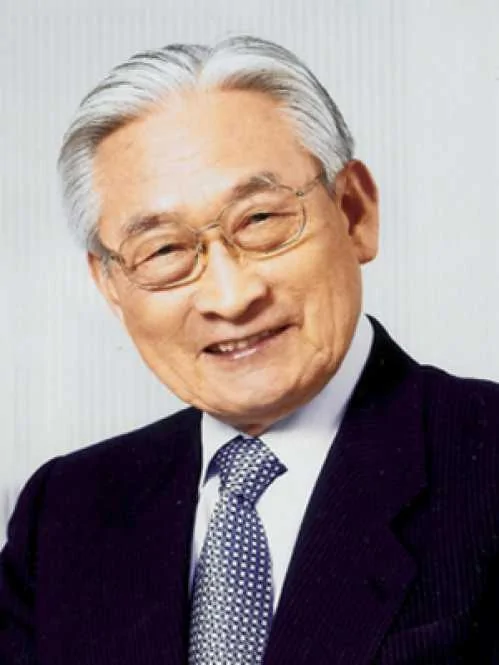

Dr Wan-Kyu Cho, one of the founding figures of modern biological science in Korea, passed away on July 13, 2026, at the age of 98. A pioneering developmental biologist, devoted educator, and visionary leader in science and higher education, Dr Cho helped establish the foundations of modern biology in Korea while playing a formative role in building the institutions and policies that enabled Korean science to flourish. Over a career spanning more than half a century, his influence extended far beyond his own research, encompassing the education of generations of scientists, the advancement of university autonomy, the promotion of biotechnology, and the internationalization of Korean science.

Born in 1928 in Jaeryong, Hwanghae Province, Dr Cho moved south after Korea’s liberation and eventually settled in Daejeon. He entered Seoul National University and received his BS in biology in 1952, followed by an MS in 1956 and a PhD in 1969. His scientific career began under extraordinarily difficult circumstances in postwar Korea, when even basic laboratory equipment and research materials were scarce. His master’s research examined the effects of antibiotics on the movement of mouse leukocytes. Because experimental resources were so limited, he subsequently turned to human genetics, including studies of the sex ratio at birth, demonstrating early in his career the resourcefulness that would characterize his scientific life.

Dr Cho joined the faculty of Seoul National University in 1957 and devoted approximately 36 years to research and education there. He later became a pioneer of developmental biology in Korea, with a particular focus on mammalian oocyte maturation and early embryonic development. Among his most influential findings was the demonstration that cyclic AMP could reversibly inhibit the meiotic maturation of mouse oocytes, work that contributed to the emerging understanding of the regulation of meiotic cell-cycle progression. He also developed the *micro-tube culture method*, an ingenious and economical technique that allowed mammalian oocytes, fertilized eggs, and embryos to be cultured and transported in small tubes. These studies exemplified his ability to turn the severe limitations of the Korean research environment of the time into opportunities for methodological innovation.

His achievements as an educator were equally profound. During his years at Seoul National University, Dr Cho published more than 100 scientific papers and trained 30 master’s and 18 doctoral students. His academic descendants came to be known collectively as the “Seollang school” (雪浪門下生), after his pen name *Seollang* (雪浪). Many of these students went on to lead biological and biomedical research at universities and research institutes in Korea and abroad, extending his influence across subsequent generations of Korean life scientists.

Dr Cho’s contribution to Korean science, however, extended far beyond the laboratory. When Seoul National University reorganized and moved to its Gwanak campus, he served as the founding dean of the College of Natural Sciences and helped establish systems that are now taken for granted at a modern research university, including open faculty recruitment, centralized management of research funds, and institutional support for basic scientific research. He subsequently served as Vice President of Seoul National University and, from 1987 to 1991, as its 18th President. Assuming the presidency during a period of profound social and political change, he promoted university autonomy and democratization. Among other measures, the university removed restrictions on students’ political activities and allowed approximately 1,000 previously disciplined students to return to their studies.

His leadership subsequently expanded to the national level. Dr Cho served as President of the Korean Federation of Science and Technology Societies, Chairman of the Presidential Advisory Council on Science and Technology, and Minister of Education. Recognizing the future importance of biotechnology well before it became a major national industry, he played a leading role in establishing the institutional framework for genetic engineering research in Korea, including promotion of the Genetic Engineering Promotion Act. He also helped establish the Korea Bioindustry Association, strengthening links between academic research and the biotechnology industry. In 1994, he became the founding President of the Korean Academy of Science and Technology, helping to create a national academy capable of representing Korean science internationally.

Another enduring achievement was his pivotal role in bringing the International Vaccine Institute (IVI) to Korea. As chairman of the Korean committee seeking to host the institute, Dr Cho led the effort that resulted in Korea being selected as the host country in 1994. He subsequently served on IVI’s founding Board of Trustees and helped establish the IVI Korea Support Committee in 1998, serving as its first chairman and later as a senior advisor. IVI has described him as one of its founders and credited his leadership, vision, and sustained commitment as instrumental in establishing in Seoul what became Korea’s first international organization headquartered in the country.

For his contributions to science, education, and public service, Dr Cho received numerous honors, including the Order of Civil Merit, Moran Medal, the Order of Service Merit, Cheongjo Medal, and the Order of Science and Technology Merit, Changjo Medal. He received the Inchon Award in Education in 2006 and was designated one of the inaugural Men of Merit in Science and Technology of the Republic of Korea in 2017. In 2023, Seoul National University selected him as a recipient of its *Proud SNU Person* award in recognition of his exceptional contributions to the university, Korean science, and education.

Remarkably, Dr Cho remained deeply engaged with science and the scientific community into his final years. Even in his nineties, he continued to attend scientific and public meetings, encourage younger researchers, and speak passionately about the future of Korean science. He often emphasized that scientific discovery should arise from curiosity and the joy of finding something new rather than merely from predetermined goals. To younger scientists, he stressed perseverance, independence, and the importance of maintaining creativity even under difficult circumstances. His hopes for Korean science remained firmly directed toward the future, and until late in life he expressed his wish to see Korean scientists reach the highest levels of international scientific achievement.

For me, Dr Cho was not only a towering figure in Korean biology but also my teacher. I had the privilege of receiving my master’s training under his guidance at Seoul National University. To those of us who belonged to the Seollang academic family, his legacy lies not only in his scientific discoveries or the many institutions he helped create, but also in the standards he set for how a scientist should think, work, and contribute to society. His career demonstrated that scientific excellence, education, institution building, and public service need not be separate pursuits but can be different expressions of the same commitment to science and to future generations.

Dr Cho will be deeply missed by his family, students, colleagues, and the entire Korean scientific community. Yet the institutions he built, the scientists he trained, and the scientific culture he helped establish remain very much alive. Few individuals have accompanied—and helped shape—the transformation of Korean science from its difficult beginnings after the Korean War to its present international standing as closely as he did. His life was, in this sense, inseparable from the history of modern Korean science. For those of us fortunate enough to have learned from him, Seollang will remain not only a distinguished scientist and leader but a teacher whose example will continue to guide us.

## CRediT authorship contribution statement

**Min Whan Jung:** Writing – review & editing.

